# International Center of Excellence for Malaria Research for South Asia and Broader Malaria Research in India

**DOI:** 10.4269/ajtmh.22-0005

**Published:** 2022-10-13

**Authors:** Anjali Mascarenhas, Rimi Chakrabarti, Laura Chery-Karschney, John White, Kristen M. Skillman, Usheer Kanjee, Prasad H. Babar, Rapatbhorn Patrapuvich, Ajeet Kumar Mohanty, Manoj T. Duraisingh, Pradipsinh K. Rathod

**Affiliations:** ^1^ Department of Chemistry, University of Washington, Seattle, Washington;; ^2^ Department of Medicine, Goa Medical College and Hospital, Bambolim, Goa, India;; ^3^ Department of Immunology and Infectious Diseases, Harvard T.H. Chan School of Public Health, Boston, Massachusetts;; ^4^ Drug Research Unit for Malaria, Center of Excellence in Malaria Research, Faculty of Tropical Medicine, Mahidol University, Bangkok, Thailand;; ^5^ National Institute of Malaria Research, New Delhi, India

## Abstract

The Malaria Evolution in South Asia (MESA) International Center of Excellence for Malaria Research (ICEMR) conducted research studies at multiple sites in India to record blood-slide positivity over time, but also to study broader aspects of the disease. From the Southwest of India (Goa) to the Northeast (Assam), the MESA-ICEMR invested in research equipment, operational capacity, and trained personnel to observe frequencies of *Plasmodium falciparum* and *Plasmodium vivax* infections, clinical presentations, treatment effectiveness, vector transmission, and reinfections. With Government of India partners, Indian and U.S. academics, and trained researchers on the ground, the MESA-ICEMR team contributes information on malaria in selected parts of India.

## INTRODUCTION

Malaria has been a recurring global health problem for centuries, with at least 29 countries currently showing malaria infections.^1^ India, one of the countries affected, has a history of taking strong measures to counter the spread of malaria. As far back as 1958, India launched its National Malaria Eradication Program, with significant successes in subsequent decades.^2^^,^^3^ Unfortunately, India experienced a resurgence of malaria beginning in the early 1970s^4^^,^^5^ that was often attributed to operational difficulties and resistance to the primary mosquito-control insecticide dichlorodiphenyltrichloroethane. The National Institute of Malaria Research (NIMR), created in 1977, and the National Vector-Borne Disease Control Program of India (now called the National Center for Vector Borne Diseases Control) subsequently created in 2003, under the umbrella of the Ministry of Health and Family Welfare, Government of India, focused additional resources to curb malaria in India. Today, with an advancing research base and a fast-growing economy, India has also funded malaria research at premier academic research institutions across the country. Government hospitals and medical colleges are receiving support to improve clinical descriptions of malaria. There is a growing interest in bridging clinical and basic science to advance even further opportunities for improving malaria control and treatment across India.^6^^,^^7^

**Figure 1.**
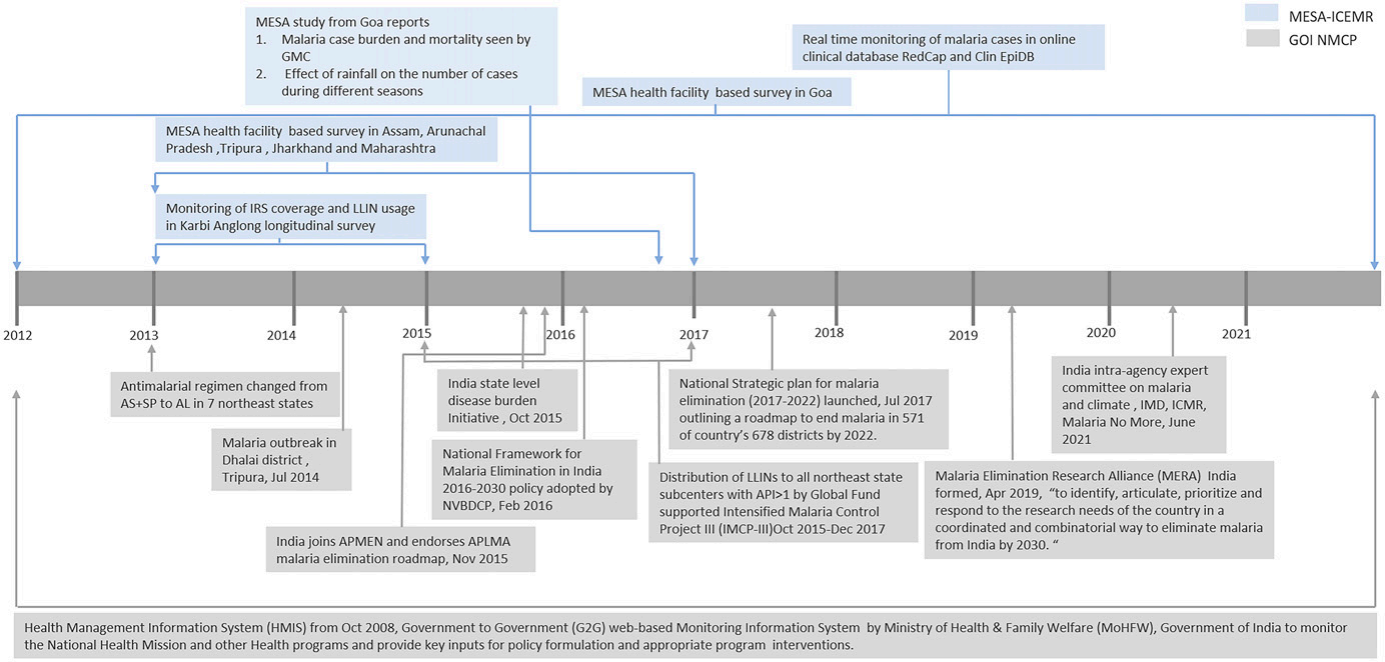
Impact of major changes in treatment and control measures policies captured by Malaria Evolution in South Asia (MESA)–International Center of Excellence for Malaria Research (ICEMR) sites between 2012 and 2021. Some of the recent Government of India National Malaria Control Program (GOI NMCP) policy initiatives, such as effect of environmental factors on malaria cases, have already been adopted and reported by MESA-ICEMR. AL = artemether–lumefantrine; API = Annual Parasite Index; APLMA = Asia Pacific Leaders Malaria Alliance; APMEN = Asia Pacific Malaria Elimination Network; AS = artesunate; GMC = Goa Medical College; ICMR = Indian Council of Medical Research; IMD = Indian Meteorological Department; IRS = indoor residual spraying; LLIN = long-lasting insecticide-treated net; NVBDCP = National Vector-Borne Disease Control Program; SP = sulfadoxine–pyrimethamine. This figure appears in color at www.ajtmh.org.

With support from the U.S. NIH, and in collaboration with the Indian Council of Medical Research, the Malaria Evolution in South Asia (MESA) International Center of Excellence for Malaria Research (ICEMR) was organized to perform basic science and clinical research in India. The goal was to assess our present understanding of malaria pathogenesis, virulence, and transmission at multiple locations across India. In this framework, the MESA-ICEMR has witnessed new and evolving Government of India initiatives and policies that have been advanced to check transmission and treatments (Figure 1). Given larger global pressures to control malaria throughout the world, in cooperation with a large number of stakeholders, India made a commitment to eliminate malaria completely by 2030. To this effect, a National Framework for Malaria Elimination in India 2016–2030 was set up with the goal of eliminating malaria within 15 years and, subsequently, to maintain a malaria-free status in the country.^8^ The framework proposes a multipronged approach, including streamlining programs that include malaria control initiatives, research strategies, surveillance, active and passive reporting of malaria cases, and monitoring of drug resistance. There remain concerns regarding fragmented communication among research entities, unintegrated results from various laboratories, ineffective reporting to the national program, and insufficient engagement by policymakers. All of this has inspired the need for a common platform to synthesize existing initiatives.

A single platform—called the Malaria Elimination Research Alliance (MERA)–India, led by scientists and clinicians within the Indian Ministry of Health, was established by the Government of India in 2019 to identify research priorities.^9^ The U.S.-based MESA-ICEMR is not formally a part of MERA-India, but is uniquely poised to cooperate with the MERA mission because some our Indian collaborators are part of MERA. MESA research today continues to have some synergistic overlap with the broad categories that MERA has defined, which include vector biology and community behavior. Upcoming MESA research, which has been delayed by the COVID pandemic, is in line with research priorities now also identified by MERA. The MESA-ICEMR team meets regularly with national and international malaria health-care experts from across the world who come as expert advisors or as collaborators. This, in turn, has led to many regular science meetings between U.S. NIH MESA-ICEMR scientists, the MESA-ICEMR partners in India, and representatives of key malaria control agencies in the Government of India. This has also facilitated the exchange of information between basic scientists and malaria control stakeholders at both the state and the national levels.

## STAKEHOLDERS AT STUDY SITES

For sampling diverse malaria cases across the country, the MESA-ICEMR team established several malaria study sites in India during the past decade. These include research laboratories at Goa Medical College (GMC), Goa; NIMR-Goa, Goa; the Regional Medical Research Center–Dibrugarh (RMRC-Dibrugarh), Dibrugarh, Assam; and field sites at Shalini Hospital–Krishi Gram Vikas Kendra, Ranchi, Jharkhand; and Acharya Vinoba Bhave Rural Hospital, Wardha. At the field sites, patients with malaria are recruited into the study, blood samples are collected, and data are recorded. The MESA-ICEMR laboratory at GMC-Goa also serves as a central site where new scientists and staff undergo training in patient recruitment and laboratory techniques, and where visiting scientists and collaborators gather for ICEMR-related scientific meetings. Moreover, research collaborations with government organizations such as NIMR-Delhi and public institutions such as the Indian Institute of Technology–Bombay, Mumbai, have also been established.

### Goa Medical College-Goa.

GMC is the only government tertiary health-care facility in a peri-urban setting in the small state of Goa. With both *Plasmodium falciparum* and *Plasmodium vivax* malaria endemic in the state, GMC treats both the local and migrant populations in large enough numbers to allow for adequate sampling of both mild and severe malaria infections of both *Plasmodium* species. In Goa, the MESA-ICEMR program was able to ascertain a demographic profile of both *P. falciparum* and *P. vivax* malaria, with diagnostic and clinical indicators, over a 4-year period.^10^ As expected, the increase in the number of positive malaria cases coincided with the onset of the rainy season. The clinical data collected, and the severity indicators noted, allow for better diagnosis in the future. Moreover, alliance with global definitions of malaria disease severity enable clinicians in India to understand epidemiology and pathogenesis of infection more completely. To gain further insight into severe malaria for India, and beyond India, the MESA-ICEMR team studied the host–receptor interactions in both *P. falciparum*– and *P. vivax*–infected red blood cells, particularly *P. falciparum* erythrocyte membrane protein 1 on the surface of infected erythrocytes. These proteins varied in their ability to bind to endothelial protein C receptors and to inhibit activated protein C– endothelial protein C receptor interactions.^11^ Together with high parasite biomass, such variations in parasite binding to blood vessels can influence disease severity.^12^

With the relatively high volume of non-severe *P. vivax* cases seen annually at the GMC, the experimental approaches used in the MESA-ICEMR research laboratory at the GMC have led to insightful observations and results. MESA scientists initially focused on comparing cryopreservation efficacy between Indian *P. vivax* isolates and previously used Brazilian isolates.^13^ The MESA-ICEMR team developed a modified version of counting (using a microscope reticle) to increase the accuracy of measuring both *P. vivax* parasitemia and reticulocytemia, which are frequently low.^14^ Even these simple advancements have improved accuracy in parasite counting of patient samples at field sites, research laboratories, and clinical settings. Previously published data from Southeast Asia showed that *P. vivax* prefers to infect reticulocytes ex vivo.^15^ Interestingly, we observed parasitemia for Indian *P. vivax* isolates that was greater than that of reticulocytemia, suggesting that Indian isolates differ in their reticulocyte preference.^16^ *Plasmodium vivax* has proved to be more challenging to culture through in vivo or even ex vivo assays compared with *P. falciparum*, mainly because *P. vivax* infects reticulocytes predominantly, which are difficult to isolate.^17^ MESA-ICEMR partners established a novel method of enrichment of *P. vivax*–infected reticulocytes and also found that *P. vivax* infection greatly decreases the osmotic stability of the infected reticulocyte.^18^^,^^19^ Studies involving *P. vivax* continue to be a high priority for the MESA-ICEMR, which has a strong interest in understanding variations in disease severity in malaria.

### NIMR-Goa.

The MESA-ICEMR laboratory at NIMR-Goa, built with a fully functional, state-of-the-art insectary, was crucial for the controlled vector infection studies conducted in India using both *P. falciparum* and *P. vivax*. In the initial work, the MESA-ICEMR team discovered a new vector, *Anopheles subpictus*, to be a contributor to perennial transmission.^20^ Membrane feeding experiments were optimized using *Anopheles stephensi* mosquitoes hatched directly from field larvae^21^ compared with earlier published work with colonized *An. stephensi* laboratory colonies.^22^^,^^23^ These experiments focused on comparison of *Plasmodium* infections induced in both field-derived and colonized *An. stephensi* mosquitoes, and subsequent sporozoite production.^21^ The MESA-ICEMR team was the first to report optimized *P. vivax* sporozoite production in *An. stephensi* at an Indian field site, and the techniques learned will be beneficial for other vector biologists both inside and outside India.^24^ Investigations involving sporozoites in *P. vivax* liver stage assays and transmission-blocking experiments are currently underway.

### Indian Institute of Technology–Bombay, Mumbai.

As a part of the ongoing coordinated effort by all ICEMRs to identify shared antibody responses to a number of parasite antigens, the MESA-ICEMR group initially teamed up with Indian Institute of Technology–Bombay, Mumbai, to determine the serological profiles for 200 Indian patients infected with either *P. falciparum* or *P. vivax*, or both. Using protein arrays, seroreactivity was measured against recombinant *P. falciparum* and *P. vivax* antigens. This study^25^ indicates that the seroreactivity for the *P. falciparum* antigen was pronounced and is comparable to the seroreactivity as seen from endemic areas such as Africa. Of 248 seropositive *P. falciparum* antigens, MSP10, heat shock protein 70, PTP5, AP2, AMA1, and SYN6 Merozoite Surface Protein 10 (MSP10), Heat Shock Protein 70 (HSP70), PfEMP1-trafficking protein (PTP5), Apetala 2 (AP2), Apical Membrane Antigen 1(AMA1), and SNARE protein (SYN6) showed strong reactivity to patient serum IgG. For *P. vivax* patient sera, however, ETRAMPs, MSPs, and ApiAP2, sexual stage antigen s16, RON3 Early Transcribed Membrane Proteins (ETRAMPs), Merozoite Surface Proteins(MSPs), Apicomplexan Apetela 2(ApiAP2), sexual stage antigen Pfs16, and Rhoptry Neck Protein 3 (RON3) were the key antigens that showed strong seroreactivity. This study^25^ also identified different antigens from severe and uncomplicated patient sera that showed strong seroreactivity. This protein array data from India, coupled with similar evidence from other NIH ICEMRs around the world, identified key antigens that could be used as a measure of exposure, especially in low-transmission settings.^25^ The MESA-ICEMR team will probe and identify additional parasite antigens that could be used for malaria diagnosis in rapid diagnostic tests, and to provide a better understanding of host–parasite interactions.^26^

### RMRC-Dibrugarh.

In addition to MESA-ICEMR studies in Goa, a second highly active partnership is with a Government of India research laboratory in RMRC (Indian Council of Medical Research), Dibrugarh, in the northeastern state of Assam. Assam and other neighboring states of India, share ∼4,000 km of international borders with surrounding countries and provide a potential route for entry of novel forms of malaria drug resistance into India.^27^ These northeastern states also harbor high levels of *P. falciparum* malaria. The hilly terrain, vast forests, tribal inhabitants, poor infrastructure, and frequent floods present challenges in terms of diagnosis and treatment of malaria infections. These environmental conditions not only hamper malaria research, they also interfere with effective malaria interventions, such as the distribution of treated bed nets and effective indoor spraying. To gain a deeper perspective on the complexity of malaria transmission in these areas, longitudinal population studies with extensive follow-up would be most informative. The MESA-ICEMR team, with partners in Assam, set up a large cohort study in the high-transmission region of the Karbi Anglong District (manuscript in preparation). The goal was to assess the impact of epidemiological and socioeconomic factors on progression of disease in individuals and households over a 2-year period. The MESA-ICEMR study observed a greater malaria prevalence in the community, a high burden of asymptomatic cases, and some delayed parasite clearance (manuscript in preparation) compared with earlier reports.^28^^,^^29^ Cohort studies to assess additional epidemiological factors in high malaria transmission settings, and investigations into delayed parasite clearance in treated individuals are currently being planned.

Dissemination of relevant evidence from northeastern India in publications, and presentations at Indian scientific meetings and symposia, have enabled the MESA-ICEMR team to bridge the gap regularly between field practices and laboratory malaria research in India. Inter-site communication within MESA-ICEMR has ensured that work is not duplicated, and continual contact between collaborators ensures that imaginative new ideas and techniques are transferred quickly from the laboratory to field sites. Continued close interactions among practicing clinicians, university medical departments, and state-level health committees remain a priority as the MESA-ICEMR team monitors malaria trends across India.

## POTENTIAL INFLUENCE ON MALARIA RESEARCH POLICIES

India is a big, populous country, and malaria in India is complex and heterogeneous. The heterogeneity includes region-specific variations in malaria transmission intensity, *P. falciparum*-to-*P. vivax* ratios, vector species distribution, and varying sensitivity to artemisinin combination therapy (ACT). This heterogeneity requires that subnational malaria control policies must be tailored to particular regions. For example, India has adopted two different first-line ACT regimens, one for Northeast India and another for the rest of India.^30^^,^^31^ To ensure an adequate strategy that is responsive to both short-term challenges (reduction in transmission burden) and long-term opportunities (malaria eradication), policymakers need an accurate description of the current malaria situation. The MESA-ICEMR research activities in India described in detail in an accompanying article (“Malaria Presentation across NIH South Asia ICEMR Sites”^32^) can provide information on the current malaria situation and thus have some influence on regional malaria control policies.

Study outcomes from both our current research and planned research can provide complementary data for evidence-based policymaking at the regional level (Table 1). The MESA-ICEMR surveillance studies constantly monitor the role of environmental (rainfall, temperature, and humidity), sociodemographic (e.g., occupation, migration), and entomological factors that influence transmission at our study sites, as well as the local impact of current malaria control measures.^10^^,^^20^^,^^33^ In addition, our basic sciences research aims to understand the mechanistic underpinnings of clinical phenotypes, both in general and specific to India. The MESA-ICEMR findings will be informative to those developing blood-stage vaccines, and to provide tools for evaluating their efficacy. The surface of the invasive merozoite form of the parasite is directly exposed to the immune system in the bloodstream, and the molecules that mediate invasion have been proposed as vaccine candidates.^34^ India is a major player in malaria vaccine development.^35^ There is no continuous-culture system for the growth of *P. vivax*, but the MESA-ICEMR team has used a collection of live patient isolates to develop invasion assays. This will aid in mapping parasite invasion ligand expression and their interactions with red blood cell receptors. Furthermore, the invasion assay platform will allow MESA-ICEMR to test directly whether potential vaccine candidates such as recombinant invasion ligands,^36^ and whether both parasite- and host-targeted antibodies, are capable of blocking invasion. For *P. vivax*, for which very little is known about invasion, the MESA-ICEMR team will continue to establish much needed methods essential to blocking invasion. Such fundamental initiatives are unachievable in other settings.

**Table 1:** Potential impact of the Malaria Evolution in South Asia (MESA)–International Center of Excellence for Malaria Research (ICEMR) in malaria control policy and development of malaria health products

MESA-ICEMR research areas overlapping with malaria core interventions	Potential impact of current and future MESA-ICEMR research in malaria control policy and developing malaria health products
1. Community engagement	Relevance of current community engagement Required improvements based on community feedback
2. Vector control	Adoption of tailor-made, region-specific vector control policy based on *Anopheles* species responsible for transmission and their seasonal distribution, biting behavior, and insecticide resistance status Expanded entomological surveillance to report on the role of current as well as previously uncharacterized vectors in regional transmission
3. Case management	Diagnosis Treatment	Addressing the potential need for improved or novel diagnostic tools Point-of-care molecular diagnostic method for low-density infections *Plasmodium vivax* hypnozoite detection markers Severe *P. vivax* malaria biomarkers Wider G6PD deficiency testing Continued evaluation of PfHRP2-based rapid diagnostic tests by monitoring PfHRP2 deletions in study populations Most effective and safe treatment policy based on antimalarial resistance and G6PD deficiency status
4. Surveillance	Region-specific transmission interventions such as targeting the most at-risk group Designing cost-effective vector control strategies based on seasonal peaks of cases Deciding on the need for active case detection or reactive case detection in a particular at-risk population based on transmission patterns

G6PD = glucose-6-phosphate dehydrogenase; PfHRP2 = *Plasmodium falciparum* histidine-rich protein 2.

Efforts to monitor ACT sensitivity at the MESA-ICEMR study sites in India are still in preliminary form. Future expansion of these research efforts will provide a more detailed view of the effectiveness of current antimalarial regimens in India, and whether the current treatment policies need to be updated.

## EMERGING RESEARCH AND IMPLICATIONS FOR FUTURE DIRECTIONS

Future research activities of the MESA-ICEMR will have an emphasis on malaria core interventions (Table 1). The cohort study in Assam (manuscript in preparation) revealed the importance of community engagement, which includes assessment of ongoing malaria prevention practices and subsequent education to prevent infections in the community. The MESA-ICEMR team will continue to assess and inform on the relevance of community engagement by the National Malaria Control Program, and identify ways to improve it based on community feedback. The MESA team along with local partners will increasingly track variables such as health-seeking behavior and LLIN use as malaria transmission decreases and more Indian states enter the elimination phase.

The MESA-ICEMR team will expand its entomological surveillance capacity, and laboratory vector studies, to include membrane feeding and parasite development studies. A high priority will be placed on poorly characterized Indian vectors that tested positive for *P. falciparum* and *P. vivax* in surveys conducted in their natural settings. This could build on earlier work pointing to poorly appreciated vectors possibly contributing to regional transmission, as we have reported previously.^20^

The MESA-ICEMR, operating in South Asia, has a strong interest in *P. vivax*, which has historically been much less explored compared with *P. falciparum.* In particular, a full understanding of the molecular basis of *P. vivax* pathogenesis is needed, and the MESA-ICEMR team aims to pursue investigations on *P. vivax* pathogenesis, red blood cell invasion, and reticulocyte tropism. *Plasmodium vivax* has been shown to cause severe malaria and deaths in South Asia, and it is important to determine how much of this is a result of particular pathogenic strains of *P. vivax* and/or a region-specific host vulnerability.^37^^–^^39^ *Plasmodium vivax*–specific mechanisms of pathogenesis remain largely unknown. The MESA-ICEMR study site in Goa at GMC has a high proportion of *P. vivax* infections (∼70% mono infections), of which ∼5% lead to severe disease manifestation. GMC thus provides an important opportunity to study questions relating to *P. vivax* pathogenesis. The major clinical complications of patients with severe *P. vivax* at GMC are anemia, jaundice, and respiratory distress.^10^ The MESA-ICEMR team will test associations of Indian *P. vivax* case data with severe disease, including parasite biomass, reticulocyte preference, endothelial activation, plasma biomarkers of coagulation, and inflammation.

The MESA-ICEMR will also explore liver-stage infections of *P. vivax*, particularly the parasite’s capacity to produce hypnozoites. Such research, in patient samples and in laboratory models, has the potential to develop diagnostic tools for identifying latent hypnozoites and to improve our understanding of relapsing infections. Improving technical approaches to understand *P. vivax* liver-stage biology is a high priority to MESA-ICEMR.

## RESEARCH CAPACITY-BUILDING AND TRAINING

The MESA-ICEMR has extensive technical resources available through its collaborators, both within India and abroad.^40^ During the past decade, the MESA-ICEMR team has worked side-by-side with Indian government institutions to broaden scientific inquiries relevant to malaria, and to do so through close engagement with affected local communities, especially hospitals and medical colleges. By building advanced laboratories in existing Indian institutions, the MESA-ICEMR helped bridge gaps between medical experts and strong basic scientists.

Even as MESA-ICEMR-mediated India–US collaborations have added fresh perspectives and innovations in malaria research for both sides, the MESA-ICEMR teams have also jointly helped build secondary expertise in statistical analysis, in novel molecular probes such as bead-based antibody detection, and in state-of-the-science insectaries. Last, the MESA-ICEMR team worked early and aggressively with the rapidly emerging biotechnology companies in India to gain high-quality, cost-effective services for primer design and DNA sequencing. This included local Indian startups and Indian branches of established companies such as Eurofins, Illumina, nanochip technology, and more. An internal, deep understanding of our questions and scientific needs allowed us to assess the quality of work outsourced in India, and set an example for others on what molecular research is possible in India, without large movements of patient samples abroad. MESA-ICEMR staff have varied educational backgrounds, with both basic and advanced science degrees from national and international educational institutions. Most newly hired staff train in laboratory techniques, including *Plasmodium falciparum* culturing, genomics, and serology, which are well established at the central MESA-ICEMR GMC site. In addition, our research scientists also have the opportunity to visit parent laboratories in Seattle (University of Washington and Seattle Children’s Hospital) or Boston (Harvard T.H. Chan School of Public Health). MESA-ICEMR scientists in India have also attended international workshops and conferences on malaria, and regularly upgrade their technical knowledge and skills.

New protocols, which include community surveys at various sites, were under the ethical board review process at most sites, but progress has been hindered as a result of the COVID-19 pandemic. We have planned community surveys in conjunction with local community members and relevant government health workers to engage cooperation among local inhabitants. We have found local involvement to be invaluable for achieving community survey objectives (unpublished data). These surveys will be region specific and will focus on assessing health-seeking behaviors, disease surveillance, presence of asymptomatic infections, and more, in the local communities.

## CONCLUSION

The MESA-ICEMR team and its Indian partners have developed infrastructure to survey, assess, and conduct malaria research in select parts of India. Although India has numerous resources to monitor national malaria control campaigns, there are components of the MESA-ICEMR activities that can add value with fresh public health questions and unique technical approaches, including deeper scientific queries into asymptomatic infections and mechanisms that initiate antimalarial resistance.

The team will also introduce administrative structures for sound and safe research. MESA-ICEMR can provide Indian authorities with an independent assessment of the malaria burden at each MESA-ICEMR study site,^8^ and can corroborate the impact of changes in Indian malaria research policy in depth at select sites (Figure 1).
